# Four Draft Single-Cell Genome Sequences of Novel, Nearly Identical *Kiritimatiellaeota* Strains Isolated from the Continental Deep Subsurface

**DOI:** 10.1128/MRA.01249-18

**Published:** 2019-03-14

**Authors:** Joshua D. Sackett, Brittany R. Kruger, Eric D. Becraft, Jessica K. Jarett, Ramunas Stepanauskas, Tanja Woyke, Duane P. Moser

**Affiliations:** a Division of Earth and Ecosystems Sciences, Desert Research Institute, Las Vegas, Nevada, USA; b Division of Hydrologic Sciences, Desert Research Institute, Las Vegas, Nevada, USA; c School of Life Sciences, University of Nevada Las Vegas, Las Vegas, Nevada, USA; d Bigelow Laboratory for Ocean Sciences, East Boothbay, Maine, USA; e Department of Biology, University of North Alabama, Florence, Alabama, USA; f Joint Genome Institute, Walnut Creek, California, USA; University of Maryland School of Medicine

## Abstract

The recently proposed bacterial phylum *Kiritimatiellaeota* represents a globally distributed monophyletic clade distinct from other members of the *Planctomycetes*, *Verrucomicrobia*, and *Chlamydiae* (PVC) superphylum. Here, we present four phylogenetically distinct single-cell genome sequences from within the *Kiritimatiellaeota* lineage sampled from deep continental subsurface aquifer fluids of the Death Valley Regional Flow System in the United States.

## ANNOUNCEMENT

Members of the recently proposed bacterial phylum *Kiritimatiellaeota* (1) (previously *Verrucomicrobia* subdivision 5 [2]) are globally distributed and found in environments such as vertebrate intestines (3), soils (4), and marine environments (1, 5, 6). However, despite their cosmopolitan distribution and prevalence in 16S rRNA gene amplicon surveys, little is known about the genomic diversity, physiology, and ecology of these organisms, particularly in deep continental subsurface environments.

To date, a single pure culture representative of the *Kiritimatiellaeota* (Kiritimatiella glycovorans L21-Fru-AB^T^), originally isolated from a hypersaline lake on the Kiritimati Atoll, has been cultivated and phenotypically and genomically characterized (1, 6). In line with previous observations of polysaccharide degradation by members of this group (5), cultivation studies and genomic analysis of K. glycovorans L21-Fru-AB^T^ suggest that this organism is saccharolytic and derives energy via fermentation (1). Here, we report four draft single-cell genome sequences representing members of the *Kiritimatiellaeota* phylum obtained from a deep, fractured rock aquifer.

Subsurface aquifer water samples were collected with a motor-driven discrete sampler from an uncased interval at a depth of 752 m below the land surface in BLM1, an 883.5-m-deep monitoring borehole drilled into Paleozoic carbonates located in Inyo County, California (36.4004°N, −116.4692°W), in August 2015. The water temperature was 57.2°C, the pH was 6.92, the electrical conductivity was 2,299 μS cm^−1^, and the oxidation-reduction potential was −242 mV. Despite a dissolved oxygen measurement of 0.43 mg liter^−1^, the downhole environment was most likely anoxic owing to its negative oxidation-reduction potential. Raw water samples (1 ml) for single-cell genomics were amended with 5% glycerol and 1× Tris-EDTA (TE) buffer (final concentrations), frozen on dry ice in the field, and stored at −80°C until cell sorting. Single cells were sorted, and their genomes were amplified and sequenced at the Bigelow Laboratory for Ocean Sciences Single Cell Genomics Center as previously described (7). Briefly, cryopreserved samples were thawed, prescreened through a 40-μm nylon mesh cell strainer (Becton Dickinson, Franklin Lakes, NJ, USA), and incubated with SYTO-9 DNA stain (Thermo Fisher Scientific, Waltham, MA, USA) at a final concentration of 5 μM for 10 to 60 min. Fluorescence-activated cell sorting was performed with a BD InFlux Mariner flow cytometer equipped with a 488-nm laser and a 70-μm nozzle orifice (Becton Dickinson). The cytometer was triggered on side scatter, and the “single-1 drop” mode was used for maximal sort purity. The sort gate was defined based on particle green fluorescence, light side scatter, and the ratio of green versus red fluorescence (for improved discrimination of cells from detrital particles). For each sample, individual cells were deposited into 384-well plates containing 600 nl per well of 1× TE buffer and stored at −80°C prior to subsequent processing. Of the 384 wells, 317 wells were dedicated for single particles, 64 wells were used as negative controls (no droplet deposition), and 3 wells received 10 particles each to serve as positive controls. Cells were lysed, and their DNA was denatured with 5 freeze-thaw cycles, the addition of 700 nl of lysis buffer (0.4 M KOH, 10 mM EDTA, and 100 mM dithiothreitol), and a subsequent 10-min incubation at 20°C. Lysis was terminated by the addition of 700 nl of 1 M Tris-HCl at pH 4.

Sequencing libraries were created for each single cell with the Nextera XT DNA library preparation kit (Illumina, San Diego, CA, USA) with the following modifications: purification was performed with column cleanup kits (Qiagen, Venlo, the Netherlands), and library selection was performed with BluePippin (Sage Science, Beverly, MA, USA) with a target sequence size of 500 ± 50 bp. Libraries were sequenced with the NextSeq 500 platform (Illumina) and V1 reagents (2 × 150-bp paired-end sequencing). Raw sequencing reads for each single amplified genome (SAG) were quality trimmed with Trimmomatic v0.32 (8), reads with 95% or greater nucleotide identity with the Homo sapiens reference genome assembly (GRCh38) were removed, and low-complexity reads (less than 5% of any nucleotide) were removed as described previously (7). Quality-filtered reads were normalized *in silico* with kmernorm 1.05 (http://sourceforge.net/projects/kmernorm) using the settings –k 21 –t 30 –c 3 and subsequently assembled into contigs with SPAdes v3.9.0 (9) with the following settings: –careful –sc –phred-offset 33. Contig ends (100 bp) were trimmed, and contigs of fewer than 2,000 bp were discarded. Genome completeness and potential contamination were estimated with CheckM v1.0.8 (10). Predicted genome size was calculated by dividing assembly size by estimated genome completeness. Assembly quality for each SAG was determined according to minimum information about single amplified genome (MISAG) standards (11). Protein-encoding regions were identified with the Rapid Annotations using Subsystems Technology (RAST) server (12), and genes were annotated with Koala (KEGG) (13) and InterProScan 5 (14). Average nucleotide identity (ANI) of reciprocal hits between genome assemblies was calculated using the online ANI calculator (http://enve-omics.ce.gatech.edu/ani/) (15). Assembly statistics are shown in Table 1.

**TABLE 1:** Assembly and quality statistics for BLM1 *Kiritimatiellaeota* SAGs

	Data for SAG:			
Assembly statistic	AH-151-K23	AH-147-K21	AH-151-C14	AH-151-A22
Raw read accession no.	SAMEA5244953	SAMEA5244950	SAMEA5244951	SAMEA5244952
Assembly accession no.	CAACVW010000000	CAACVX010000000	CAACVZ010000000	CAACVY010000000
Annotation accession no.	3300022259	3300022272	3300022292	3300022301
No. of raw paired-end reads	9,035,693	4,110,878	8,955,047	7,063,659
No. of quality-filtered paired-end reads	222,128	312,138	731,454	1,107,044
Assembly size (bp)	431,650	688,295	1,629,841	2,632,675
G+C content (%)	61.5	61.6	61.8	62.1
Estimated genome completeness (%)^*a*^	3.4	17.4	38.9	69.0
Predicted genome size (Mbp)^*a*^	12.52	3.95	4.19	3.82
Estimated contamination (%)^*a*^	0	0.7	0.9	0
Genome quality^*b*^	Low	Low	Low	Medium
No. of contigs	73	54	161	137
Largest contig (bp)	30,074	56,953	91,734	150,914
*N*_50_ value	7,761	20,490	16,267	37,970
No. of protein-coding genes	409	595	1,394	2,191
No. of tRNA genes	5	15	25	39
No. of rRNA genes	4	3	3	3

a Estimated with CheckM v1.0.8 (10).

b Genome quality reported according to Bowers et al. (11).

Based on the detection of conserved single-copy marker genes in the 3 most complete SAG assemblies, we predict that BLM1 *Kiritimatiellaeota* genome sequences contain 3.8 to 4.2 Mbp. The CheckM-based predicted genome size of the smallest SAG (AH-151-K23) was 3 times higher than values for the other SAGs. CheckM estimates genome completeness and contamination of genome assemblies based on the presence and location of lineage-specific marker genes selected from the phylogenetic placement (based on single-copy marker genes in the assembly) of the assembly in a built-in reference genome tree (10). Of the 104 marker genes used by CheckM to assess genome completeness and contamination for AH-151-K23, only 2 genes were found in the assembly (threonylcarbamoyl adenosine biosynthesis protein TsaE [accession no. PF02367] and Holliday junction DNA helicase RuvA [accession no. TIGR00084]), ultimately resulting in 3.4% estimated genome completeness. Furthermore, compared to the other SAGs, the largest contig size (30 kb) and *N*_50_ value (7.7 kb) associated with AH-151-K23 were ∼2 to 5 times lower. A combination of the absence of phylogenetically informative marker genes in the assembly, low genome recovery (small assembly), and relatively short contigs contributed to very low genome completeness and high genome size predictions for this SAG.

All four SAGs had identical 16S rRNA genes and shared greater than 99% average nucleotide identity. The 16S rRNA gene sequence has 82.7% sequence identity with K. glycovorans L21-Fru-AB^T^ (GenBank accession no. KC665948) (1), suggesting that these 2 organisms belong to genetically distinct lineages. The SAGs encode a variety of glycosyl hydrolases, including cellulases (GH5), β-xylosidases (GH39), d-4,5-unsaturated β-glucuronyl hydrolases (GH88), glucoamylases (GH97), and endo-α-*N*-acetylgalactosaminidases (GH101) as well as many uncharacterized sulfatases. These results suggest that these organisms may have the capacity for degradation of complex polysaccharides and glycoproteins to obtain carbon, amino acids, and sulfur, as has been previously suggested for members of this phylum (1). Comprehensive reconstruction of the metabolic pathways encoded in the SAGs will further deepen our understanding of the ecology of these unique *Kiritimatiellaeota* strains in the deep continental subsurface.

### Data availability.

Raw sequencing reads and genome assemblies for the four SAGs have been deposited in the EMBL ENA under project no. PRJEB30981. SAG-specific accession numbers are listed in Table 1. Annotations have been deposited in the Joint Genome Institute’s Integrated Microbial Genomes and Microbiomes database (JGI IMG/M) under the accession numbers listed in Table 1.
